# Alternative Splicing of *TCF7L2* Gene in Omental and Subcutaneous Adipose Tissue and Risk of Type 2 Diabetes

**DOI:** 10.1371/journal.pone.0007231

**Published:** 2009-09-30

**Authors:** Ludmila Prokunina-Olsson, Lee M. Kaplan, Eric E. Schadt, Francis S. Collins

**Affiliations:** 1 Laboratory of Translational Genomics, Division of Cancer Epidemiology and Genetics, National Cancer Institute, National Institutes of Health, Bethesda, Maryland, United States of America; 2 Massachusetts General Hospital (MGH) Weight Center, Boston, Massachusetts, United States of America; 3 Pacific Biosciences, Menlo Park, California, United States of America; 4 Genome Technology Branch, National Human Genome Research Institute, National Institutes of Health, Bethesda, Maryland, United States of America; Uppsala University, Sweden

## Abstract

**Background:**

Single nucleotide polymorphisms (SNPs) rs7903146 and rs12255372 located within *TCF7L2* gene have been identified as the strongest common genetic risk factors for development of type 2 diabetes (T2D). We hypothesized that these genetic variants might increase the risk of T2D through regulation of alternative splicing or expression level of *TCF7L2* in human adipose tissue.

**Methodology/Principal Findings:**

Expression of 13 assays detecting alternatively spliced forms of *TCF7L2* was measured by quantitative reverse-transcriptase PCR (qRT-PCR) in paired biopsies of omental and subcutaneous adipose tissue from 159 obese individuals (BMI 54.6+/−12.2 kg/m^2^). *TCF7L2* expression in both types of adipose tissue was not associated with SNPs rs7903146 and rs12255372, T2D status and blood levels of glucose or glycosylated hemoglobin (HbA1c). Expression of assays “ex12-13”, “ex12-14” and “ex13-13a” detecting C-terminal alternative exons of *TCF7L2* was higher in subcutaneous compared to omental adipose tissue by 1.46 fold (p = 6.5×10^−15^), 1.41 fold (p = 1.4×10^−9^) and 1.26 fold (p = 4.7×10^−6^) in the control group and by 1.86 fold (p = 1.7×10^−4^), 1.77 fold (p = 7.3×10^−4^) and 1.58 fold (p = 6.1×10^−4^) in the T2D group. A pathway enrichment analysis on transcripts significantly co-expressed with *TCF7L2* in a microarray set combined with individual expression assays, suggested tissue-specific roles of *TCF7L2* splicing forms in regulation of transcription, signal transduction and cell adhesion.

**Conclusions:**

Expression of *TCF7L2* alternatively spliced forms may have different functional roles in omental and subcutaneous adipose tissue but is not associated with SNPs rs7903146 and rs12255372 or T2D status.

## Introduction

Common intronic single nucleotide polymorphisms (SNPs) within the transcription factor 7 - like 2 gene (*TCF7L2*) have been identified as genetic factors that significantly increase risk of type 2 diabetes (T2D) [1], [2], [3], [4]. *TCF7L2* belongs to a family of TCF/LEF transcription factors that interact with β-catenin and regulate the WNT pathway [5]. Activation of the WNT pathway leads to increased cell proliferation due to effects of downstream targets of *TCF7L2* such as *MYC* [6], [7] and *CCND1* (Cyclin D1) [8]. A complex interplay of activation and repression of the WNT pathway, orchestrated by different protein isoforms of TCF/LEF transcription factors, is required for tissue-specific differentiation of stem cells. For example, differentiation of skin stem cells into either hair follicle or sebum-producing cells is regulated by expression of alternatively spliced forms of the LEF1 transcription factor [9]. Similarly, an active WNT pathway is required for myogenesis, while inactivation of the pathway by a dominant-negative form of *TCF7L2* promotes adipogenesis [10]. Increased adiposity, as measured by body mass index (BMI), is a strong risk factor for development of insulin resistance, T2D and cardiovascular disease [11]. Several studies have reported that patients carrying risk alleles of the associated SNPs rs7903146 and rs12255372 of *TCF7L2* have lower BMI compared to carriers of non-risk alleles [12], [13], [14], [15]. Potentially, risk alleles of *TCF7L2* might increase risk of T2D even in lean individuals, or affect diabetes and adiposity through independent mechanisms.

Non-coding genetic variants can affect mRNA expression and splicing [16], [17]. Several studies attempted to correlate genotypes of T2D-associated variants of *TCF7L2* with mRNA expression of *TCF7L2* in adipose tissue [13], [18] skeletal muscle [18], lymphoblastoid cell lines [18] and pancreatic islets [19], [20], but no consistent associations have been reported. One study detected a significant decrease in *TCF7L2* expression in obese individuals with T2D compared to obese controls, but this study was based only on six samples [13]. We previously catalogued and evaluated expression of multiple splicing forms of *TCF7L2* in several types of human tissue [21]. We observed tentative association between expression of several assays for C-terminal exons of *TCF7L2* and genotypes of SNPs rs7903146 and rs12255372 in pancreatic islets but not in a small set of samples of subcutaneous adipose tissue [21].

Here, we used 13 assays detecting all known splicing forms of *TCF7L2* to evaluate gene expression in paired biopsies of subcutaneous and omental adipose tissue from 159 obese individuals. We evaluated the association between expression of these assays and genotypes of T2D-associated variants rs7903146 and rs12255372, T2D status, type of adipose tissue, BMI (37.6–89.6 kg/m2) and blood levels of glucose and glycosylated hemoglobin (HbA1c). We show that expression of alternatively spliced forms of *TCF7L2* may have different functional roles in omental and subcutaneous adipose tissue but is not associated with SNPs rs7903146 and rs12255372 or T2D status.

## Results

### Characteristics of the T2D and control groups

**Figure 1:**
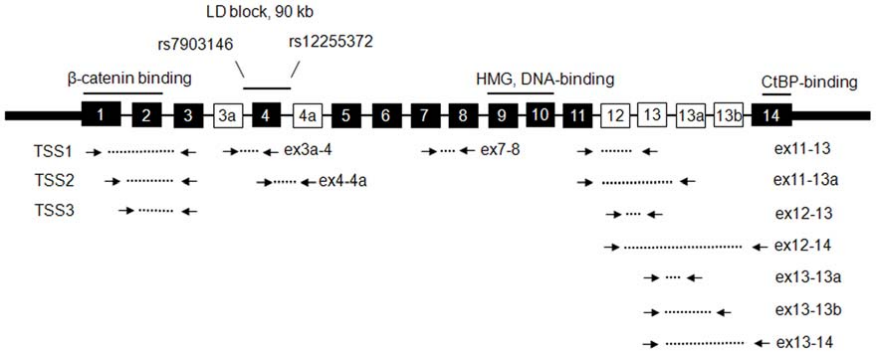
Structure of *TCF7L2* gene and location of expression assays. The scheme shows constitutive exons (black rectangles), alternative exons (white rectangles); linkage disequilibrium (LD) block with associated SNPs rs7903146 and rs12255372; protein domains: ß-catenin-interacting domain, DNA-binding HMG domain and C-terminal Binding Protein (CtBP)-binding domain; expression assays for detection of alternative splicing forms of *TCF7L2* are indicated by connected arrows: assays TSS1, TSS2 and TSS3 target transcripts produced from alternative transcription start sites while other assays target alternatively spliced forms with different combinations of exons, * - expression of assay “ex13-13b” was tested but not detected in adipose tissue.

SNPs rs7903146 and rs12255372 are located in introns 3 and 4 of *TCF7L2* gene within the associated linkage disequilibrium (LD) block and 50 kb apart from each other [1], [2], [3] (Fig. 1). In our set of 159 Caucasian individuals, the frequencies of risk alleles of both SNPs were higher in the T2D group (n = 16) than in the control group (n = 143), 0.41 in T2D vs. 0.26 in controls for rs7903146 and 0.38 in T2D vs. 0.25 in controls for rs12255372 (Table 1). Similarly to other European sets [1], [2], [3] and to the European set (CEU) of the HapMap [22], these two SNPs were in high linkage disequilibrium (LD) with each other (D′ = 0.88, r^2^ = 0.73). Samples in the T2D and control groups were matched by age, gender and BMI by design but several T2D-related traits were significantly different between these groups: levels of blood glucose, HbA1c and homeostasis model of insulin resistance (Homa-IR) were increased, while the level of HDL cholesterol was decreased in the T2D group (Table 1).

**Table 1:** Characteristics of the T2D and control groups.

Trait	T2D Mean (st.dev)	T2D N	Control Mean (st.dev)	Control N	p-value*
Age, years	46.53 (12.42)	16	43.78 (10.04)	143	0.30
Male: female ratio	52.9∶47.1	16	47.6∶52.4	143	0.68
BMI, Kg/m2	53.62 (15.77)	16	54.77 (11.73)	143	0.49
Cholesterol, mg/dl	167.0 (31.48)	15	189.98 (34.70)	130	0.0091
Triglycerides, mg/dl	205.73 (114.73)	14	168.69 (91.53)	129	0.18
Leptin, mg/dl	43.5 (27.58)	2	58.11 (31.19)	36	NA
LDL, mg/dl	93.6 (32.8)	14	110.77 (29.42)	127	0.016
HDL, mg/dl	37.19 (9.93)	15	46.63(11.65)	130	0.0004
WBC, ×10^9^ cell/l	9.16 (2.55)	15	8.41 (2.11)	139	0.22
Insulin, mg/dl	32.73 (25.53)	8	23.15 (14.16)	102	0.077
Glucose, mg/dl	197.82 (51.19)	16	98.04 (14.25)	143	2.04×10^−37^
Homa-IR	316.21 (320.98)	8	102.11 (70.95)	102	5.58×10^−7^
HbA1C	8.24 (1.30)	16	5.79 (0.59)	119	1.15×10^−23^
Rs 7903146		32		268	0.097
C	0.594		0.738		
T	0.401		0.262		
Rs12255372		32		286	0.14
G	0.625		0.748		
T	0.375		0.252		

* Two-sided T-test or Chi-square test (for allele frequencies) not adjusted for covariates and multiple tests.

### Test for association between *TCF7L2* expression and genotypes of rs7903146 and rs12255372, T2D status and blood levels of glucose and HbA1c

Levels of expression of 13 *TCF7L2* assays in both types of adipose tissue were adjusted for age, sex, BMI and blood levels of glucose and HbA1c. Expression of these assays in each individual tissue and the ratio of subcutaneous: omental expression was similar in the control and T2D groups and in carriers of different genotypes of SNPs rs7903146 and rs1225532 (Table 2 and 3). Expression of assay “ex13-13b” (Fig. 1) previously studied in human pancreatic islets [21] was tested but not detected in adipose tissue (data not shown). Based on the number of samples and the observed standard deviation in expression of each assay, we had 80% power and 95% confidence to detect a >1.2-fold difference in expression between groups with risk and non-risk alleles for each SNP and between the T2D and control groups.

**Table 2:** Expression of *TCF7L2* in omental and subcutaneous adipose tissues: comparison between T2D and control groups, 119 controls, 16 T2D patients.

Expression assay	Ratio^a^ SA∶OA p-value^b^	OA p-value^b^	Effect in T2D^c^	SA p-value^b^	Effect in T2D^c^
TSS1	0.054	0.130	+	0.327	+
TSS2	0.069	0.074	+	0.057	+
TSS3	0.050	0.064	+	0.052	+
Ex3a-4	0.106	0.054	−	0.128	+
Ex4-4a	0.200	0.141	+	0.067	+
Ex7-8	0.103	0.050	−	0.150	+
Ex11-13	0.074	0.095	+	0.290	+
Ex11-13a	0.151	0.078	+	0.430	+
Ex12-13	0.194	0.116	−	0.098	+
Ex13-13a	0.185	0.051	−	0.277	+
Ex12-14	0.054	0.055	−	0.051	+
Ex11-14	0.409	0.755	−	0.052	+
Ex13-14	0.134	0.051	−	0.197	+

SA-subcutaneous adipose, OA- omental adipose.

a in paired samples of subcutaneous and omental adipose tissue.

b p-values for univariate analysis, adjusted for age, sex, BMI and blood levels of glucose and HbA1c but not adjusted for multiple tests.

c increase (+) or decrease (−) in expression in T2D group compared to controls.

**Table 3:** Effect of presence 0, 1 or 2 risk alleles of *TCF7L2* SNPs on expression of *TCF7L2* in paired omental and subcutaneous adipose tissue samples, n = 134.

Expression assay	OA p-value^b^ rs7903146	SA p-value^b^ rs7903146	Ratio^a^ p-value^b^ rs7903146	OA p-value^b^ rs12255372	SA p-value^b^ rs12255372	Ratio^a^ p-value^b^ rs12255372
TSS1	0.368	0.168	0.341	0.288	0.306	0.186
TSS2	0.176	0.157	0.216	0.277	0.162	0.128
TSS3	0.166	0.170	0.185	0.143	0.533	0.358
Ex3a-4	0.075	0.081	0.079	0.072	0.114	0.064
Ex4-4a	0.203	0.069	0.204	0.064	0.064	0.109
Ex7-8	0.188	0.126	0.254	0.218	0.051	0.128
Ex11-13	0.269	0.070	0.285	0.221	0.068	0.147
Ex11-13a	0.122	0.331	0.485	0.217	0.274	0.583
Ex12-13	0.348	0.050	0.255	0.303	0.071	0.148
Ex13-13a	0.256	0.088	0.252	0.315	0.097	0.261
Ex12-14	0.137	0.055	0.111	0.174	0.133	0.056
Ex11-14	0.410	0.071	0.217	0.421	0.114	0.116
Ex13-14	0.187	0.085	0.135	0.178	0.102	0.066

SA-subcutaneous adipose, OA- omental adipose.

a ratio subcutaneous∶omental expression in paired samples.

b p-value for linear regression model with 0, 1 or 2 risk alleles of *TCF7L2* SNPs adjusted for age, sex, BMI, T2D status and blood levels of glucose and HbA1c but not adjusted for multiple tests.

### Difference in *TCF7L2* expression between omental and subcutaneous adipose tissues

**Figure 2:**
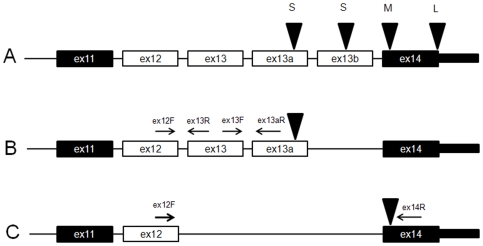
Alternative splicing forms of *TCF7L2* with increased expression in subcutaneous compared to omental adipose. A. Constitutive exons 11 and 14 are marked as black rectangles, alternative exons 12, 13, 13a, 13b are marked as white rectangles, black triangles above exons indicate location of alternative stop codons that define protein reading frames: short (S), medium (M) or long (L); B. Alternative splicing form with exons 11-12-13-13a (GenBank FJ010174) has an alternative stop codon within exon 13a and encodes a protein with short reading frame; C. Alternative splicing form with exons 11-12-14 (GenBank FJ010170) has an alternative stop codon in the beginning of exon 14 and encodes a protein with medium reading frame. Positions of expression assays “ex12-13”, “ex13-13a” and “12-14” used in this study are indicated above corresponding exons. Assays “ex12-13”, “ex13-13a” and “12-14” detect all splicing forms of *TCF7L2* that include alternative exon 12.

Expression of several assays was higher in subcutaneous compared to omental adipose tissue both in the control and T2D groups: 1.46 and 1.86-fold for assay “ex12-13”, 1.41 and 1.77-fold for assay “ex12-14” and 1.26 and 1.58-fold for assay “ex13-13a” (Table 4). Only expression of alternative exon 3a (assay “ex3a-4”) was decreased in subcutaneous compared to adipose tissue (0.81 fold) in the control group, but not in the T2D group (0.91 fold) (Table 4). The results for assays “ex12-13”, “13-13a” and “12-14” will remain significant even after adjustment for multiple tests. Different ways of normalization of *TCF7L2* expression (by expression levels of endogenous controls *B2M*, *GAPDH* or both genes together) did not significantly affect the conclusions (data not shown). The assays “ex12-13”, “13-13a” and “12-14” detect two distinct splicing forms that include alternative exon 12 (Figure 2). The first form (GenBank FJ010174) includes C-terminal exons 11-12-13-13a and encodes a protein with a short reading frame terminated by an alternative stop codon within exon 13a. The second form (GenBank FJ010170) includes exons 11-12-14 and encodes a protein with a medium reading frame terminated by an alternative stop codon in the beginning of exon 14 (Fig. 2).

**Table 4:** Expression of *TCF7L2* in omental and subcutaneous adipose tissue in controls (n = 143) and T2D (n = 16) groups.

Expression assay	Controls, OA, reference	Controls, SA, fold to reference	controls p-value*	T2D OA, reference	T2D SA, fold to reference	T2D p-value*
TSS1	1.0	1.06	0.20	1.0	1.13	0.29
TSS2	1.0	1.04	0.539	1.0	1.23	0.283
TSS3	1.0	1.00	0.066	1.0	1.20	0.353
Ex3a-4	1.0	0.81	2.6×10^−4^	1.0	0.91	0.516
Ex4-4a	1.0	1.07	0.231	1.0	1.21	0.185
Ex7-8	1.0	1.08	0.047	1.0	1.24	0.056
Ex11-13	1.0	1.09	0.053	1.0	1.16	0.213
Ex11-13a	1.0	1.09	0.068	1.0	1.13	0.0013
Ex12-13	1.0	1.46	6.45×10^−15^	1.0	1.86	1.7×10^−4^
Ex13-13a	1.0	1.26	4.7×10^−6^	1.0	1.58	6.1×10^−4^
Ex12-14	1.0	1.41	1.4×10^−9^	1.0	1.77	7.3×10^−4^
Ex11-14	1.0	1.23	2.7×10^−4^	1.0	1.49	0.0120
Ex13-14	1.0	1.13	0.0062	1.0	1.32	0.052

SA-subcutaneous adipose, OA- omental adipose.

* - Two-sided T-test, p-values are not adjusted for multiple tests; expression in omental adipose is taken as 1.0 for each assay.

### Pathway enrichment analysis of *TCF7L2* expression

To better understand the function of *TCF7L2* in human adipose tissue we searched for transcripts that positively or negatively correlated with expression of *TCF7L2*. We combined global expression data measured by microarrays [23] with data for individual expression assays “ex7-8”, “ex3a-4”, “ex12-13” and “ex13-13a”, generated in the same set of 159 samples of omental and subcutaneous adipose tissue. On the microarrays, *TCF7L2* was represented by 2 probes located within the 3′ untranslated region (UTR) of RefSeq transcript NM_030756. The microarray probes and the assay “ex7-8” detect a mix of all splicing forms of *TCF7L2*, while the assays “ex12-13”, “ex13-13a” and “ex3a-4” detect distinct splicing forms that showed difference in expression between the two types of adipose tissue. There was a significant correlation between the microarray and TaqMan expression data in omental adipose tissue: r2 = 0.51 for assay “ex7-8”, r2 = 0.49 for assay “ex13-13a”, r2 = 0.42 for assay “ex12-13” and r2 = 0.31 for “ex3a-4” but in subcutaneous adipose tissue the correlation for the same assays was not significant (r2<0.25). Using the joint expression set (microarrays and TaqMan data), we identified sets of transcripts that correlated with expression of these assays at a cutoff of r2>+/−0.25, corresponding to False Discovery Rate (FDR) p<0.0015, and analyzed each set of transcripts for enrichment of Gene Ontology categories (GO). In omental adipose tissue *TCF7L2* expression positively correlated with activation of transcription through KRAB box Zn finger transcription factors but negatively correlated with protein biosynthesis, signal transduction, oxidative phosphorylation and electron transport (Table 5). In subcutaneous adipose tissue the strongest positive correlation was between expression of assay “ex13-13a” and the cadherin signaling pathway and cell adhesion, while negative correlation was detected with protein biosynthesis and receptor function (Table 5). Full list of transcripts with positive and negative correlations with *TCF7L2* assays “ex12-13” and “ex13-13a” are presented in Tables S2, S3, S4, S5.

**Table 5:** Panther Classification System analysis of *TCF7L2* expression in adipose tissue.

Gene Ontology category	Tissue, correlation^a^	*TCF7L2* assay	array, n^b^	observed, n^c^	expected, n^d^	direction^e^	p-value^f^
Ribosomal protein	OA, N	ex3a-4	473	31	5.15	+	3.23E-13
Protein biosynthesis	OA, N	ex3a-4	591	31	6.43	+	9.73E-11
Oxidative phosphorylation	OA, N	ex3a-4	85	13	.92	+	2.62E-09
Signal transduction	OA, N	ex3a-4	3412	10	37.12	−	6.33E-07
Electron transport	OA, N	ex3a-4	254	15	2.76	+	5.44E-06
Defense/immunity protein	OA, N	ex12-13	401	13	3.32	+	9.87E-04
Nucleic acid metabolism	OA, P	ex13-13a	3038	105	70.58	+	5.10E-04
Transcription factor	OA, P	ex13-13a	1796	71	41.73	+	2.41E-04
KRAB box transcription factor	OA, P	ex7-8	431	65	22.04	+	8.01E-12
Nucleic acid metabolism	OA, P	ex7-8	3038	233	155.37	+	3.51E-09
Zinc finger transcription factor	OA, P	ex7-8	727	80	37.18	+	4.92E-08
Cell surface receptor mediated signal transduction	OA, P	ex7-8	1596	36	81.62	−	5.52E-07
Receptor	OA, P	ex7-8	1476	33	75.49	−	3.51E-07
G-protein mediated signaling	OA, P	ex7-8	786	12	40.20	−	2.42E-05
Ribosomal protein	SA, N	ex3a-4	473	45	19.44	+	5.48E-05
Receptor	SA, N	ex12-13	444	42	18.20	+	2.81E-05
Cadherin	SA, P	ex13-13a	156	21	2.23	+	4.17E-12
Cadherin signaling pathway	SA, P	ex13-13a	214	21	3.05	+	1.57E-09
WNT signaling pathway	SA, P	ex13-13a	400	25	5.71	+	1.96E-07
Cell adhesion-mediated signaling	SA, P	ex13-13a	433	24	6.18	+	4.88E-06

OA – omental adipose; SA – subcutaneous adipose; P-positive correlation with *TCF7L2*, N-negative correlation with *TCF7L2*.

a transcripts with positive or negative correlation (r2>+/−0.25, p<0.0015) with expression of *TCF7L2* assays.

b number of transcripts in each GO category among 20,316 annotated transcripts on the array.

c number of transcripts with significant correlation with *TCF7L2* expression in each GO category.

d expected number of transcripts in each GO category based on the frequencies on the array.

e direction (enrichment or deficit) in each GO category.

f p-value for differences between observed and expected number of transcripts in each GO category Bonferroni-adjusted for number of GO categories.

## Discussion

Our study provides a detailed analysis of expression of multiple *TCF7L2* splicing forms in paired biopsies of omental and subcutaneous adipose tissue from a large set of obese individuals. The first goal of this study was to evaluate the association between the expression of *TCF7L2* splicing forms and T2D-associated SNPs rs7903146 and rs12255372, T2D status or blood levels of glucose or HbA1c. Despite 80% of power to detect >1.2 fold differences in expression between different groups, we did not observe any significant association between these factors. Our results are similar to a recently published data on 235 samples of subcutaneous adipose tissue where expression of *TCF7L2* (measured by one assay corresponding to our “ex7-8” assay) was not affected by T2D status, sex, and genotypes of rs7903146 [24].

The second goal of this study was to compare the expression of *TCF7L2* splicing forms in two types of adipose tissue. We observed that expression of assays “ex12-13”, “ex12-14” and “ex13-13a” was higher in subcutaneous compared to omental tissue and this difference could not be explained by genotypes of the T2D-associated SNPs, sex, BMI and blood levels of glucose and HbA1c. These findings were similar to the results of a study that did not detect association between expression of assay “ex9-10” of *TCF7L2* and genotypes of rs7903146, rs12255372, age, sex and BMI in paired biopsies of adipose tissue from 49 individuals [25]. However, the expression of this assay was 4.8-fold higher in subcutaneous compared to omental tissue in controls and 6-fold higher in the T2D group [25]. The effects observed in our study were weaker, 1.46-fold in the control group and 1.86-fold in the T2D group and the effect was not detectable for assay “ex7-8” that measures the same splicing forms as assay “ex9-10”. This could potentially be attributed to the difference in sample size (159 samples in our study and 49 samples in Kovacs et al.), use of different custom-designed expression assays and reference genes (one reference gene at Kovacs et al. and two reference genes in our study). Additionally, individuals in our study were more obese (BMI 54.6+/−12.2) compared to less obese individuals in Kovacs et al (BMI 29.7+/−0.8 kg/m2) [25].

*TCF7L2* is a ubiquitously expressed transcription factor that mediates signals of the WNT pathway. The function of *TCF7L2* and, particularly, in different types of adipose tissue is not clear. A pathway enrichment analysis on transcripts significantly coexpressed with *TCF7L2* showed that the main role of *TCF7L2* in omental adipose tissue is associated with activation of transcription and inhibition of protein biosynthesis and signal transduction. In contrast, in subcutaneous adipose tissue we did not observe any evidence for correlation with activation of transcription, but with increased cell adhesion through cadherin protein family (for assay “ex13-13a”). Adipose consists of several types of cells such as adipocytes, endothelial cells, macrophages that can differently express *TCF7L2*. For example, assays “ex12-13” and “13-13a” specifically detect a unique splicing form that includes exons 12-13-13a, expressed in multiple tissues and is the major *TCF7L2* splicing form in peripheral blood monocytes ([21], GenBank FJ010174, Figure 2). Adhesion of monocytes to endothelial lining of capillaries, infiltration into adipose tissue and differentiation into macrophages contributes to low-grade chronic inflammation in adipose tissue and development of insulin resistance [20]–[22]. Therefore, increased expression of assays “ex12-13” and “ex13-13a” in subcutaneous adipose tissue and co-expression with genes involved in cell adhesion might indicate increased monocytes influx into subcutaneous adipose tissue or other functional differences between these two tissue types. All assays of *TCF7L2* with significantly increased expression in subcutaneous compared to omental adipose (“ex12-13”, “ex13-13a” and “ex12-14”) detect splicing forms that encode truncated TCF7L2 protein with short or medium reading frames and include an alternative exon 12 (Figure 2). The truncated protein isoforms of TCF7L2 lack binding sites for the CTBP protein are not post-translationally regulated and may provide alternative regulation of the WNT pathway [26].

In conclusion, we did not observe association between expression of *TCF7L2* splicing forms, T2D status and genotypes of T2D-associated SNPs rs7903146 and rs12255372. Differential expression of *TCF7L2* splicing forms with alternative exon 12 encoding for truncated protein isoforms of TCF7L2 in omental and subcutaneous adipose tissues deserves future studies.

## Materials and Methods

### Ethics Statement

The study on anonymized samples was approved by the Internal Review Board (IRB) of Massachusetts General Hospital, protocol #2001-P-001942/22 and exempted from IRB approval at the NHGRI/NIH.

### Tissue samples

Paired samples of omental and subcutaneous adipose tissue were obtained from bariatric patients that underwent weight-reduction surgery and signed informed consent forms [23]. Following traits were available for all or subsets of samples: age, gender, BMI, white blood cells counts (WBC), fasting levels of glucose, insulin, total cholesterol, HDL and LDL cholesterol, triglycerides, homeostasis model of insulin resistance (Homa-IR) and glycosylated hemoglobin (HbA1c). The T2D status was assigned to individuals with fasting blood glucose higher than 140 mg/dl (7.8 mmol/L). Total RNA from flash-frozen adipose samples was extracted using Trizol reagent (Invitrogen) and DNA was isolated from liver samples of the same patients with DNeasy kit (Qiagen).

### Genotyping

Genotyping was performed with pre-developed TaqMan allelic discrimination assays for rs7903146 and rs12255372 (Applied Biosystems). The genotyping success was above 99% and only samples genotyped for both markers were used.

### Quantitative reverse-transcriptase PCR (qRT-PCR) expression studies

cDNA was prepared from total RNA of selected samples of omental and subcutaneous adipose tissue used for the microarray expression studies [23]. For each sample, 100 ng of total RNA was convereted to cDNA with SuperScript III reverse transcriptase and random hexamers (Invitrogen). Expression assays for *TCF7L2* were custom designed for each splicing form ([21] and Table S1). Primers for SYBR Green assays were purchased from Integrated DNA Technologies and TaqMan assays were manufactured on demand by Applied Biosystems. qRT-PCR was performed in a 10 ul reaction volume in 384 well plates with Power SYBR Green master mix (Applied Biosystems) or Expression Master Mix for TaqMan assays (Applied Biosystems) on the Sequence Detection System 7900 (Applied Biosystems). Each expression assay was run in technical duplicates and the average values for each sample were normalized to a geometric mean of mRNA levels of reference genes beta 2 microglobulin (B2M, assay Hs00187842_m1, Applied Biosystems) and Glyceraldehyde 3-phosphate dehydrogenase (GAPDH, assay 4333764F, Applied Biosystems) run in separate reactions from the same cDNA preparations.

### Statistical analyses

qRT-PCR expression was measured in Ct values (PCR cycle at which expression was detected). For each sample and assay, the average of technical replicates was first normalized to a geometric mean values of reference genes, B2M and GAPDH according to the formula: Ct(geometric) = (Ct(B2M)+Ct(GAPDH))/2. Relative expression of each assay was calculated as: dCt = Ct(geometric)−Ct(assay). For each assay, the dCt values were first tested for normality of distribution. The association between expression of *TCF7L2* assays and the counts of risk alleles (0, 1, 2) of the T2D-associated SNPs rs7903145 and rs12255372 was tested with univariate linear regression model with adjustment for covariates (age, sex, and log-transformed values of BMI and blood levels of glucose and HbA1c). The analyses were performed with SPSS 16.0 (SPSS Inc.). Microarray expression was analyzed with R statistical programming package. Power analysis was performed with StatMate 2.0 (GraphPad) and was based on standard deviations in expression of each of assays, number of samples with 0 or 1 and 2 risk alleles at each SNP and 80% power to detect the difference with 5% of type I error.

### Joint analysis of the microarray and TaqMan data, pathway enrichment analysis

The microarray data for 159 paired samples of omental and subcutaneous adipose tissue was a part of a previously described study [23]. The microarray data was available to all authors of the article and is available upon request. Each of the RNA samples was profiled with custom-designed microarrays that included 39,280 oligonucleotide probes representing 34,266 known and predicted genes (Agilent Technologies, Palo Alto, CA). We used assays “ex3a-4”, “ex7-8”, ex”12-13” and “ex13-13a” of *TCF7L2* for joint analysis of expression. Expression of *B2M* and *GAPDH* was used as an endogenous control for TaqMan *TCF7L2* expression. However, when we combined the TaqMan and microarray data, we observed that the sets of transcripts that correlated with *TCF7L2* expression significantly overlapped with the set of transcripts that correlated with *GAPDH* and *B2M* expression. To remove this biologically relevant variation component from the *GAPDH* and *B2M* expression, we identified a set of transcripts that correlated with the geometric mean of *B2M* and *GAPDH* expression at a 5% FDR (false-discovery rate). We then performed Principal Component Analysis on this set of transcripts to identify those factors (eigenvectors) that explained greater than 90% of the variation in the *GAPDH/B2M* geometric mean and adjusted the *GAPDH/B2M* expression by each of these eigenvectors. The adjusted *B2M/GAPDH* expression was then used to normalize *TCF7L2* expression using standard linear regression methods. For co-expression analysis, the normalized expression values for all transcripts were adjusted for age and sex and the residuals were computed using the rlm function from the R statistical package (M-estimation with Tukey's bisquare weights). The Spearman correlation coefficients for expression of *TCF7L2* and all other transcripts were calculated. The FDR for these correlations was calculated by performing 1000 permutations of sample IDs while preserving the correlation structure among the gene expression values. The threshold for Spearman coefficient was set up at r = +/−0.25, corresponding to FDR<1% (p-value<0.0005). The pathway enrichment analysis on sets of transcripts with significant correlations was performed using the Panther Classification System [27]. For each GO category, we compared expected and observed frequencies between a set of 20,316 annotated transcripts presented on the array and the sets of transcripts significantly correlated with *TCF7L2* expression. The p-value for significance of enrichment in each group was adjusted for the number of GO categories using Bonferroni method implemented in the Panther analysis [27].

## Supporting Information

Table S1 Primers and probes for expression assays.
(0.04 MB RTF)

Table S2 List of significant correlations between TCF7L2 assay.
(0.05 MB XLS)

Table S3 List of significant correlations between TCF7L2 assay.
(0.27 MB XLS)

Table S4 List of significant correlations between TCF7L2 assay.
(0.17 MB XLS)

Table S5 List of significant correlations between TCF7L2 assay.
(0.11 MB XLS)
