# Supplementary material for: Alternative Splicing of TCF7L2 Gene in Omental and Subcutaneous Adipose Tissue and Risk of Type 2 Diabetes
Source: PLoS One. 2009 Sep 30;4(9):e7231. doi: 10.1371/journal.pone.0007231 (PMC2747626; doi:10.1371/journal.pone.0007231)
Supplement: Table S1 — Primers and probes for expression assays. (0.04 MB RTF) [file pone.0007231.s001.doc]

| **Table S1.** Primers and probes for expression assays | | |
| --- | --- | --- |
| Expression assays, Type | Primers, probes or commercial assays from Applied Biosystems | Amplicon  size, bp |
| TCF7L2,TSS1  SYBR Green | P1ex1F: GGTGGAGGGGATGACCTA  Ex3R: GGGATCATGATGAAGGGGTA | 307 bp |
| TCF7L2, TSS2  SYBR Green | P2ex1F: AAACGAATCAAAACAGCTCCT  Ex3R: GGGATCATGATGAAGGGGTA | 168 bp |
| TCF7L2, TSS3  SYBR Green | P2intr1F: TTCTTTTTCTCCCCCTTCTCC  Ex3R: GGGATCATGATGAAGGGGTA | 211 bp |
| TCF7L2, ex3a-4  SYBR Green | Ex3aF: ACTCTGCGTACAAAACGATTGA  Ex4R: AGCAGTGGCCATTTCATCTG | 66 bp |
| TCF7L2, ex4a  SYBR Green | Ex4F: GGCCACTGCTTGATGTCC  Ex4aR: TGAGTGCTGACAGTGAAGTCTG | 162 bp |
| TCF7L2, ex7-8,  TaqMan | Hs00181036_m1 |  |
| TCF7L2,  ex11-13, TaqMan | Ex11F: GAAGAAGAGGAAAAGGGACAAGCA  Ex13R: GCGCTCGGCATTTCTTAGGA  FAM-probe: CAGGTCATTGGTCTCTCC | 70 bp |
| TCF7L2,  ex11-13a, TaqMan | Ex11F: GAAGAAGAGGAAAAGGGACAAGCA  Ex13aR: CGGTCAAGCCCGAACAGT  FAM-probe: ACCAATGATGCAAATACT | 86 bp |
| TCF7L2,  ex11-14, TaqMan | Ex11F: CGCGGGATAACTATGGAAAGAAGAA  Ex14R: CTTGTATGTAGCGAACGCACTTTT  FAM-probe: TTTTCTCATTGGTCTCTCCCGGCTG | 94 bp |
| TCF7L2,  ex12-13, TaqMan | Ex12F: ACACAGCGAATGTTTCCTAAATCCT  Ex13R: CGCGCTCGGCATTTCTTAG  FAM-probe: TTCCTCCGATTACAGACCTG | 80 bp |
| TCF7L2,  ex13-13a, TaqMan | Ex13F: GCTTTGGCCTTGATCAACAGAATAA  Ex13aR: CGGTCAAGCCCGAACAGT  FAM-probe: TTGCAGATGCAAATACT | 88 bp |
| TCF7L2,  ex12-14, TaqMan | Ex12F: ACACAGCGAATGTTTCCTAAATCCT  Ex14R: CTTGTATGTAGCGAACGCACTTTT  FAM-probe: CTCCGATTACAGGAGAAAA | 57 bp |
| TCF7L2,  ex13-14, TaqMan | Ex13F: GCTTTGGCCTTGATCAACAGAATAA  Ex14R: CTTGTATGTAGCGAACGCACTTTT  FAM-probe: TCTCCTGCAAGGGCC | 74 bp |
| TCF7L2,  ex13-13b, TaqMan | Ex13F: GCTTTGGCCTTGATCAACAGAATAA  Ex13bR: CGAACGCACTTTTTTTTTCTCCATT  FAM-probe: CCCTTGCAGTCTTTG | 88 bp |
| GAPDH | 4333764F |  |
| B2M | HS00173470_m1 |  |
